# The Efficacy of Stinging Nettle (Urtica Dioica) in Patients with Benign Prostatic Hyperplasia: A Randomized Double-Blind Study in 100 Patients

**DOI:** 10.5812/ircmj.2386

**Published:** 2013-01-05

**Authors:** Alireza Ghorbanibirgani, Ali Khalili, Laleh Zamani

**Affiliations:** 1 Department of Nursing and Midwifery, Gachsaran Branch, Islamic Azad University, Gachsaran, IR Iran

**Keywords:** Stinging Nettle, Urtica Dioica, Prostatic Hyperplasia

Dear Editor,

Benign Prostatic Hyperplasia (BPH) is a common disease observed in 90 percent of men over 60 (1). Benign prostatic hyperplasia is the most common benign tumor in men (2). The exact cause of this disease is still unknown, and environmental and genetic causes are thought to be involved in its development (3). As studies show, 50 percent of men between the ages of 51 to 60 and 90 percent of men over 80 have been histologically diagnosed with BPH (4). One of the most commonly used herbal remedies is nettle, which causes anti-inflammatory, anti-tumor, antiviral effects, modulating of immune system, and relieves the symptoms of benign prostatic hyperplasia due to the compounds it contains such as phytosterols, lignans and polysaccharides (5). In a clinical trial, 287 BPH patients who had been treated with nettle (Urtica dioica) showed significant reduction in IPSS, serum PSA and prostate size (6). Researchers decided to carry out a new research aiming to investigate the efficacy of nettle on the reduction of clinical symptoms of BPH. The present study is randomized double-blind clinical trial which has been performed to investigate the effect of nettle herb in reducing symptoms of benign prostatic hyperplasia in the patients referred to the Urology Department of Imam Khomeini and Golestan Hospitals in Ahvaz, during the period of May 1 to August 1, 2011. One-hundred BPH patients were bound to having certain criteria to be included in the study; for example, they had to be between 40 to 80 years with no specific complications such as acute urinary retention, renal infection or renal failure. They were also asked to sign the consent form. They were divided randomly and double-blind into two groups of fifty. After the patients were examined by urologists and their cases of BPH were approved, they were included in the study. One group was given nettle and other group placebo (two capsules of 300mg each, 2 times a day) similarly and consistently for a period of 8 weeks. Placebo and nettle were provided in identical capsules by Goldaru Company. A checklist containing demographic information and International Prostate Symptom Score (IPSS) or AUA was used to collect data. Nine visits were made (for each patient) before entering the study and at the end of each week until the eighth week of treatment, and at the end of treatment period, the data from before, during and after the treatment were collected through the checklist. Statistical analyses were performed using the SPSS Statistical Package (Version 17.0, Chicago, IL, USA). The results are shown as mean ± standard deviation (SD). Then, general linear model repeated measures test was used to assess the difference after using drugs. A p value less than 0.05 were considered significant. The patients were also asked to inform the researchers if it they felt or noticed side effects of any sort. Provisions of the Declaration of Helsinki were observed during the procedures of the research. After an 8-week treatment, all patients completed the study. The patients’ average age was 62.4 ± 1.2. 71.2 percent had no family history, and 27.2 percent were smokers. After two months, patients were assessed in terms of clinical symptoms of BPH using AUA Score. No significant relationship was observed between demographic information and BPH symptoms in either of the groups. The results from comparison of averages of AUA scale showed that significant difference in case group (the group on nettle) before and after using the medicine, but no statistically significant difference was observed in control group (the group on placebo). The average score of AUA scale in the group taking nettle was assessed 26.511 ± 0.264 and 2.118 ± 0.431 (P = 0.000) before and after respectively, which was statistically significant, but the average score of AUA scale in the group taking placebo was determined 27.854 ± 0.744 and 27.853 ± 0.766 respectively (P = 1.000) which was not statistically significant (Table 1). At the beginning of the study, there was no statistically significant difference between the average score of AUA scale in both groups (P = 0.764). No side effects were reported by the patients in the end of the study. According to the results, nettle had a better effect in relieving clinical symptoms in BPH patients compared to placebo. Herbal medicines such as nettle have been used in many studies to treat prostate disease, and desirable results have been achieved in this regard. In three clinical trials on BPH patients, nettle had a better impact in reducing patients’ clinical symptoms than placebo (6-8). As a whole, nettle is recommended to be used more in treatment of BPH patients, given its beneficial effects in reducing BPH patients’ symptoms and its safety in terms of its side effects and its being better accepted on the side of patients. This study was supported by a grant from Azad University of Gachsaran, Iran. We are also grateful to the staff of the teaching and research center of Islamic Azad University, Gachsaran, Iran for their help in the course of this study.

**Table 1:** Comparison of Severity of Patient's Symptoms in Two Groups of Patients Receiving Stinging Nettle or Placebo during the Course of Disease

Date	Stinging Nettle, Mean ± SD	Placebo, Mean ± SD	PValue
**Before treatment**	26/511 ± 0/264	27/854 ± 0/744	0/764
**First week**	23/554 ± 0/354	27/854 ± 0/744	0/000
**Second Week**	20/454 ± 0/854	27/855 ± 0/745	0/000
**Third week**	17/654 ± 0/694	27/856 ± 0/743	0/000
**Forth week**	14/754 ± 0/974	27/854 ± 0/744	0/000
**Fifth week**	11/954 ± 0/574	27/852 ± 0/741	0/000
**Sixth week**	7/254 ± 0/864	27/858 ± 0/742	0/000
**Seventh week**	4/054 ± 0/234	27/855 ± 0/745	0/000
**Eight week**	2/118 ± 0/431	27/853 ± 0/766	0/000
